# Plasmogamic Paternal Contributions to Early Zygotic Development in Flowering Plants

**DOI:** 10.3389/fpls.2020.00871

**Published:** 2020-06-19

**Authors:** Yukinosuke Ohnishi, Tomokazu Kawashima

**Affiliations:** ^1^Kihara Institute for Biological Research, Yokohama City University, Yokohama, Japan; ^2^Department of Plant and Soil Sciences, University of Kentucky, Lexington, KY, United States

**Keywords:** plasmogamy, karyogamy, cell elongation, asymmetric division, paternal parent-of-origin effects

## Abstract

Flowering plant zygotes possess complete developmental potency, and the mixture of male and female genetic and cytosolic materials in the zygote is a trigger to initiate embryo development. Plasmogamy, the fusion of the gamete cytoplasms, facilitates the cellular dynamics of the zygote. In the last decade, mutant analyses, live cell imaging-based observations, and direct observations of fertilized egg cells by *in vitro* fusion of isolated gametes have accelerated our understanding of the post-plasmogamic events in flowering plants including cell wall formation, gamete nuclear migration and fusion, and zygotic cell elongation and asymmetric division. Especially, it has become more evident that paternal parent-of-origin effects, via sperm cytoplasm contents, not only control canonical early zygotic development, but also activate a biparental signaling pathway critical for cell fate determination after the first cell division. Here, we summarize the plasmogamic paternal contributions via the entry of sperm contents during/after fertilization in flowering plants.

## Introduction

The fusion of male and female gametes initiates the development of the next generation in sexual reproduction. In flowering plants, two sperm cells are delivered via a pollen tube to a female gametophyte. Each fuses with a female gamete, the egg cell and central cell. These two gamete fusion processes (double fertilization) result in a zygote and a primary endosperm cell, respectively. The zygote develops into an embryo that inherits the genomic information from both parents for the next generation. The primary endosperm cell develops into the endosperm that nourishes the developing embryo or seedling after germination (Dresselhaus et al., 2016).

**FIGURE 1:**
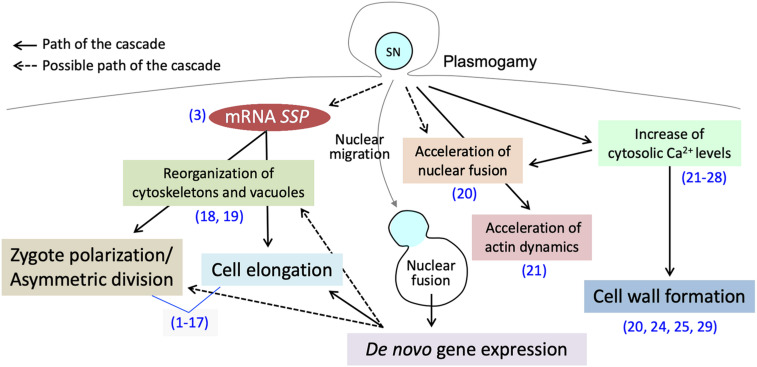
Post-plasmogamicevents in flowering plant zygotes. Paternal materials brought by the fusion of male andfemale gamete cytoplasms initiate and/or facilitate the post-plasmogamic events in flowering plant zygotes. The numbers in parentheses indicate the reference which describes the step of the pathway; (1) Lukowitz et al., 2004; (2) Wang et al., 2007; (3) Bayer et al., 2009; (4) Ueda et al., 2011; (5) Jeong et al., 2011; (6) Waki et al., 2011; (7) Fiume and Fletcher, 2012; (8) Babu et al., 2013; (9) Costa et al., 2014; (10) Huang et al., 2014; (11) Yu et al., 2016; (12) Ueda et al., 2017; (13) Yuan et al., 2017; (14) Armenta-Medina et al., 2017; (15) Zhang et al., 2017; (16) Neu et al., 2019; (17) Zhao et al., 2019; (18) Kimata et al., 2016; (19) Kimata et al., 2019; (20) Ohnishi et al., 2019; (21) Ohnishi and Okamoto, 2017; (22) Tirlapur et al., 1995; (23) Digonnet et al., 1997; (24) Antoine et al., 2000; (25) Antoine et al., 2001; (26) Hamamura et al., 2014; (27) Denninger et al., 2014; (28) Ponya et al., 2014; (29) Kranz et al., 1995.

In flowering plants, the cytosolic and genetic mixture, through the cell membrane fusion between the sperm cell and egg cell (plasmogamy) and gamete nuclear migration and fusion (karyogamy), is one of the main transitions of the life cycle (gametophytic-to-sporophytic transition) (Kawashima and Berger, 2014). Although the location of fertilization in flowering plants makes it difficult to observe such events, confocal microscopy live-cell imaging as well as direct investigation of isolated zygotes has successfully revealed intracellular dynamics during early zygote development (Kurihara et al., 2013). Direct investigation of isolated zygotes in monocots (Wang et al., 2006; Kranz and Scholten, 2008), and in eudicots (He et al., 2007; Zhao et al., 2019) is now possible. Furthermore, several *in vitro* fertilization systems have been established, allowing us further understanding of complex, yet well-orchestrated, early zygotic development (Wang et al., 2006; Kranz and Scholten, 2008; Maryenti et al., 2019). Interestingly, the studies supported by these improved observation techniques have shown much evidence that the sperm cytoplasm contributes to the post-plasmogamic events (Figure 1). In this review, we summarize the plasmogamic paternal contributions to early zygotic development in flowering plants. We also elucidate the relationship between mRNAs carried in from the sperm cell and signaling pathways controlling cell elongation and asymmetric cell division in *Arabidopsis* zygotes. Epigenetic reprogramming including imprinting is another dynamic process during sexual plant reproduction and reviewed elsewhere (Kawashima and Berger, 2014; Batista and Kohler, 2020).

## Plasmogamic Paternal Contributions Through the Increase of Cytosolic Ca^2+^ Level by Sperm Entry

In animals, the phospholipase C (PLC) isoform, PLC-zeta (PLCζ), is delivered from the sperm to oocyte, triggering cytosolic Ca^2+^ oscillations (Saunders et al., 2002; Nomikos et al., 2017). The acute rise of the cytosolic Ca^2+^ concentration plays important roles in the early zygotic development of most animals, referred to as the “egg activation” or “oocyte activation” (Stricker, 1999). The cytosolic Ca^2+^ oscillations in mammalian zygotes prevent polyspermy by inducing cortical granule exocytosis. The oscillations then activate calmodulin-dependent protein kinase II (CAMKII), indirectly triggering meiotic resumption and ultimately leading to the formation of pronuclei and entry into interphase of the first cell cycle (Sanders and Swann, 2016). The activated CAMKII promotes specific maternal mRNA degradation in *Drosophila melanogaster* zygotes, maternal protein degradation in *Xenopus*, and the degradation of both in mice (Krauchunas and Wolfner, 2013).

In flowering plants, the transient increase of the cytosolic Ca^2+^ level in the zygote immediately after sperm entry was visualized by treatment with fluorescent Ca^2+^ indicators, Fluo-3 AM and Calcium green-1, during maize and rice *in vitro* fertilization (Digonnet et al., 1997; Antoine et al., 2001; Ohnishi and Okamoto, 2017), and by a Ca^+2^-selective vibrating electrode during maize *in vitro* fertilization (Antoine et al., 2000). Using the Ca^2+^ probes Yellow Cameleon 3.60 or CerTN-L15, fluorescence resonance energy transfer probes of cytosolic Ca^2+^ that are concentration-dependent indicators, the transient increase of the cytosolic Ca^2+^ level was also observed in semi *in vivo* fertilized egg cells in *Arabidopsis* (Denninger et al., 2014; Hamamura et al., 2014). The increased Ca^2+^ level in plant zygotes induces cell wall formation (Kranz et al., 1995; Antoine et al., 2000; Ohnishi et al., 2019) and the acceleration of nuclear fusion in rice zygotes (Ohnishi et al., 2019). However, the molecular entities responsible for the increase of the cytosolic Ca^2+^ level and the molecular mechanisms of how Ca^2+^ levels control cell wall formation and accelerate the gamete nuclear fusion remain unclear. Further investigations should be performed to dissect the cytosolic Ca^2+^ increase and cellular dynamics in plant zygotes.

## A Possible Plasmogamic Paternal Contribution Enhancing Sperm Nuclear Migration

Although the molecular mechanism is still largely unknown, several factors responsible for filamentous actin (F-actin) dynamics that control sperm nuclear migration in flowering plants have been recently identified (Kawashima et al., 2014; Ohnishi et al., 2014; Ohnishi and Okamoto, 2017; Peng et al., 2017). F-actin, arranged in a network in rice egg cells, continuously converges toward the central egg nucleus and mediates the migration of the sperm nucleus (Ohnishi and Okamoto, 2017). Similar F-actin dynamics were observed in *Arabidopsis* central cells and primary endosperm cells (Kawashima et al., 2014; Kawashima and Berger, 2015), implying a common mechanism for the distinctive F-actin dynamics between the egg cell and central cell, and also between monocots and eudicots. In the rice zygote, the velocity of F-actin convergence is accelerated after sperm entry, likely supporting rapid sperm nuclear migration (Ohnishi and Okamoto, 2017). The acceleration of F-actin convergence was not observed in isolated egg cells cultured with 5 mM CaCl_2_ (Ohnishi et al., 2019). This CaCl_2_ concentration is sufficient to increase the Ca^2+^ level in the egg cytoplasm, suggesting that the Ca^2+^ increase by sperm entry itself is not enough to cause the acceleration of F-actin convergence, and there should be an additional, unidentified, pathway controlling this velocity shift. Although the molecular mechanism thereof and its biological significance are not clear, plasmogamic paternal contributions cloud enhance this early zygotic event.

## Asymmetric Division and Cell Elongation in *Arabidopsis* Zygotes

**FIGURE 2:**
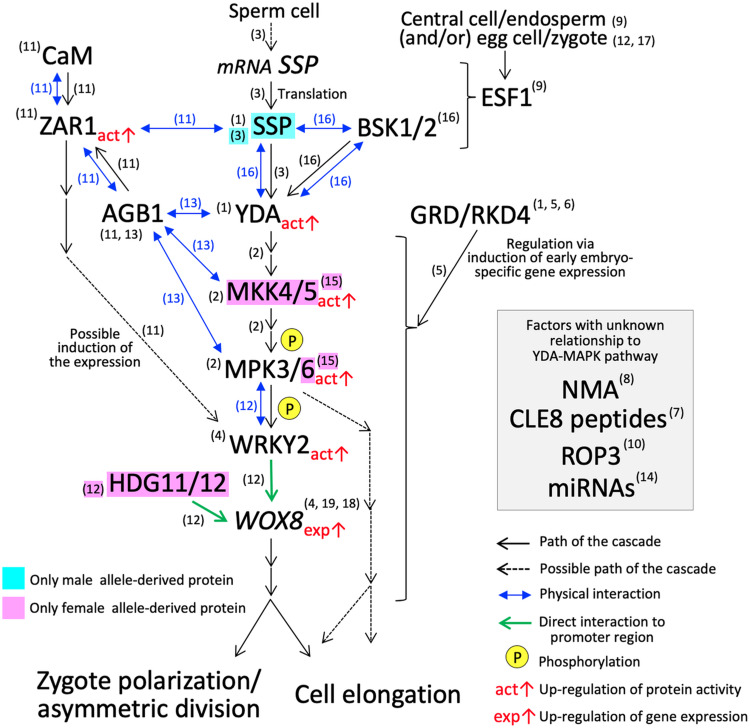
YDA-MAP cascade responsible for the cell elongation and asymmetric cell division in *Arabidopsis* zygote. The numbers in parentheses indicate the reference which describes the step of the pathway; (1) Lukowitz et al., 2004; (2) Wang et al., 2007; (3) Bayer et al., 2009; (4) Ueda et al., 2011; (5) Jeong et al., 2011; (6) Waki et al., 2011; (7) Fiume and Fletcher, 2012; (8) Babu et al., 2013; (9) Costa et al., 2014; (10) Huang et al., 2014; (11) Yu et al., 2016; (12) Ueda et al., 2017; (13) Yuan et al., 2017; (14) Armenta-Medina et al., 2017; (15) Zhang et al., 2017; (16) Neu et al., 2019; (17) Bayer et al., 2017; (18) Haecker et al., 2004; (19) Breuninger et al., 2008.

While knowledge is still sparse, several key players controlling cellular polarity that leads to asymmetric division and cell elongation of *Arabidopsis* zygotes have emerged in the last decade (Figure 2). Asymmetrical division and cell elongation of the zygote is the first hallmark of apical-basal polarity. Signaling through the YODA (YDA) mitogen-activated protein kinase kinase kinase (MAPKKK) cascade sets up the apical-basal polarity in the zygote, promoting basal-cell-lineage (suspensor) differentiation (Lukowitz et al., 2004; Wang et al., 2007). In *Arabidopsis* zygotes, YDA functions directly or indirectly as an activator of MKK4/MKK5, which subsequently activates MPK3/MPK6 by phosphorylation (Wang et al., 2007; Zhang et al., 2017). The phosphorylated MPK3/MPK6 directly activates WRKY2, a transcription factor, by phosphorylation (Ueda et al., 2017). Thereafter, WRKY2 and transcription factors, HOMEODOMAIN GLABROUS 11/12 (HDG11/12), bind directly to the *WUSCHEL RELATED HOMEOBOX 8* (*WOX8*) intron and activate its transcription in the zygote (Ueda et al., 2017). *WOX8* expression continues in the basal daughter cell after the first cell division and persists in the suspensor. Although the direct target genes remain unknown, WOX8 functions as a master regulator of zygote polarization and embryo patterning (Haecker et al., 2004; Breuninger et al., 2008; Ueda et al., 2011).

*Arabidopsis* Gβ protein (AGB1) has a direct physical interaction with MPK3/MPK6, MKK4/MKK5, and YDA, and may function as a scaffold to improve the progression of the YDA-MAPK cascade (Yuan et al., 2017). The physical interaction of AGB1 *in vitro* and *in vivo* was also demonstrated with a member of the leucine-rich repeat receptor-like kinase family, ZYGOTIC ARREST 1 (ZAR1) (Yu et al., 2016). ZAR1 also interacts with calmodulin (CaM) at the plasma membrane, and ZAR1 kinase activity is activated by the binding of CaM and/or AGB1 (Yu et al., 2016). The *zar1* catalytic (*zar1-1*) and *null* (*zar1-2*) mutations cause asymmetric division arrest and show downregulation and ectopic expression of *WRKY2* and *WOX8* in early embryo development, respectively, (Yu et al., 2016). These results suggest that ZAR1, possibly activated though a Ca^2+^ signaling cascade, has an important role in the precise progression of asymmetric division of the zygote and the subsequent embryo development. ZAR1 orchestrates the expression of genes related to the YDA-MAPK cascade. However, the direct/downstream targets remain unclear and additional verifications of this pathway especially physical interactions of factors are awaited.

*CLAVATA3/EMBRYO SURROUNDING REGION-RELATED 8* (*CLE8*), encoding small post translationally modified peptides (Fiume and Fletcher, 2012) and *Rho-like small G protein 3* (ROP3) (Huang et al., 2014) also affect *WOX8* expression pattern and/or asymmetric division. The direct/downstream targets of these factors, again, remain unknown. EMBRYO SURROUNDING FACTOR 1 (ESF1) peptides, a member of the small cysteine-rich peptide family, were first thought to be expressed specifically in the central cell/endosperm, and may be delivered into the zygote to control upstream the YDA-MAPK cascade (Costa et al., 2014). However, *ESF1* transcripts are also present in the egg cell, zygote, and early embryo (Le et al., 2010; Nodine and Bartel, 2012; Slane et al., 2014; Zhao et al., 2019), and the possible involvement of *ESF1* expressed from the maternal allele in the egg cell and/or zygote has also been pointed out (Bayer et al., 2017; Ueda et al., 2017). A mutant of *NIMNA* (*NMA*), encoding a polygalacturonase, appears to show a strong defect in zygote cell elongation, but not in the asymmetric division (Babu et al., 2013). Likewise, mutants of factors associated with zygote cell elongation and asymmetric division show a diverse range of phenotypes compared to *yda*, which shows the most severe phenotype (Supplementary Table S1).

Two-photon confocal microscopy, Kimata et al. (2016, 2019) revealed the precise dynamics of F-actin and microtubules in *Arabidopsis* zygotes. High-resolution observation with cytoskeleton inhibiter treatments indicated that the organization of microtubules plays a major role in zygote elongation (Kimata et al., 2016). For asymmetric division, nuclear positioning toward the apical end of the cell is regulated by longitudinal F-actin along the apical–basal axis and the redistribution and shape of the vacuoles (Kimata et al., 2016, 2019). The structural and kinetic mechanisms of the asymmetric division and cell elongation are gradually being elucidated, leading to further understanding of the roles of previously identified factors as well as their relationships to the YDA-MAPK cascade.

## Parent-Of-Origin Effects in Asymmetric Division and Cell Elongation of *Arabidopsis* Zygotes

In flowering plants, *de novo* transcripts from both paternal and maternal chromosomes in the zygote are immediately detectable after fertilization (Anderson et al., 2017; Chen et al., 2017; Rahman et al., 2019; Zhao et al., 2019). The inhibition of RNA polymerase II, important for *de novo* transcription, showed delay or arrest of the cell division of *Arabidopsis* zygotes (Pillot et al., 2010; Kao and Nodine, 2019; Zhao et al., 2019). Consistently, *WOX8* becomes highly expressed after fertilization (Haecker et al., 2004; Breuninger et al., 2008; Ueda et al., 2011). Parallel to the YDA-MAPK cascade, GROUNDED (GRD)/RKD4, an RWP-RK motif-containing transcription factor, activates early embryo-specific genes, which may also be downstream of the YDA-MAPK cascade (Figure 2; Lukowitz et al., 2004; Jeong et al., 2011; Waki et al., 2011). Thus, the *de novo* gene expression in the zygote is a key biological process, contributing to the subsequent zygotic developmental processes including the asymmetric division and cell elongation.

However, the majority of transcripts found in plant zygote are already present in the egg cell (Anderson et al., 2017; Chen et al., 2017; Zhao et al., 2019), indicating that maternal contribution is also critical for the onset of development (Armenta-Medina and Gillmor, 2019). For example, except *MKK5*, *WOX9*, *ZAR1*, and *CLE8*, the previously identified factors responsible for the asymmetric division and cell elongation in *Arabidopsis* are already expressed in the egg cell prior to fertilization (Zhao et al., 2019). In addition, some paternally inherited gene alleles may become activated later than the maternally inherited alleles (Del Toro-De Leon et al., 2014; Baroux and Grossniklaus, 2015; Zhao and Sun, 2015). The reciprocal cross between wild-type and each loss-of-function mutant of *MKK4/MKK5* and *MPK6* showed only a maternal effect in the asymmetric division and cell elongation (Zhang et al., 2017). *HDG11/12* genes expressed in the egg cell or from the maternal allele in the zygote are responsible for their function (Ueda et al., 2017). These findings indicate that maternal contribution is crucial for the YDA-MAPK cascade in *Arabidopsis* zygote.

On the other hand, transcriptomic analyses among gametes and zygotes also identified a group of genes that are prevalent in the sperm cell and zygote (Anderson et al., 2017; Chen et al., 2017; Rahman et al., 2019), elucidating a possible large-scale transcript carryover from the sperm cell to the zygote. A membrane-associated pseudokinase called SHORT SUSPENSOR (SSP) is known, as a paternal factor, to activate YDA (Bayer et al., 2009). *SSP* belongs to a recently diverged Brassicaceae-specific member of the *BRASSINOSTEROID SIGNALING KINASE* (*BSK*) family (Liu and Adams, 2010). Premature stop (*ssp-1*) or *null* (*ssp-2*) mutations showed symmetric division and arrested cell elongation in the zygote; however, the *ssp-2* phenotype in the Colombia-0 ecotype is much weaker than the *ssp-1* phenotype and the *yda* phenotype in the Landsberg *erecta* ecotype (Supplementary Table S1; Lukowitz et al., 2004; Bayer et al., 2009). Although the *BSK* family members, *BSK1* and *BSK2*, cannot completely complement the loss of *SSP*, these may weakly activate the YDA-MAPK cascade in parallel to *SSP* (Neu et al., 2019). The *bsk1;bsk2;ssp* triple mutant phenotype, however, is still weaker than that of *yda* (Neu et al., 2019), indicating another signaling input for the full activation of YDA.

The reciprocal cross between wild-type and *ssp* showed only a paternal effect (Bayer et al., 2009). Interestingly, *SSP* transcripts accumulate specifically in sperm cells, but are translationally repressed and SSP protein is transiently produced in the zygote only after fertilization (Bayer et al., 2009). Therefore, *SSP* transcripts are considered to have been brought in the zygote from the sperm cell via plasmogamy (Musielak and Bayer, 2014; Jeong et al., 2016; Bayer et al., 2017; Armenta-Medina and Gillmor, 2019; Wu and Zheng, 2019). However, the mechanisms of *SSP* translational repression in sperm cells and the resurgence of the translation of mRNAs carried into the zygote, still remains unclear. In animal cells, many studies of the translational regulation mediated by RNA binding proteins have been conducted (Harvey et al., 2018; Hentze et al., 2018). In plants, recent studies have gradually revealed the role of translational repression mediated by RNA-induced silencing complexes (RISCs) consisting of microRNAs (miRNAs) and ARGONAUTE (AGO) (Hou et al., 2019). AGO1-RISCs *in vitro* repress translation of the target gene, which contains sequences nearly complementary to the miRNAs (mismatches in the center of the sequence). When this sequence is located at the 5′ UTR or the ORF, translational repression occurs through blocking recruitment or movement of ribosomes (Iwakawa and Tomari, 2013). It is becoming evident that small RNAs accumulate in the pollen (Chambers and Shuai, 2009; Grant-Downton et al., 2009; Slotkin et al., 2009). Transposable element (TE) small interfering RNAs (siRNAs) participate in both post-transcriptional silencing and RNA-directed DNA methylation (Nuthikattu et al., 2013; McCue et al., 2015). They exert an effect on RNA silencing activity in sperm cells during pollen development, suggesting the transportation of TE siRNAs from the pollen vegetative cell to sperm cells (Slotkin et al., 2009; Martinez et al., 2016). Although the function of miRNA biogenesis enzyme DICER-LIKE 1 (DCL1) in zygotic development remains largely unknown and *dcl* mutants show phenotypic variability, *dcl1-7* showed arrested asymmetric division in zygotes (Armenta-Medina et al., 2017). These facts imply that the *SSP* transcripts should be under translational repression by RISCs, and that repression needs to be released after plasmogamy.

It is unclear whether the translational repression by RISCs in flowering plants is reversible, as reported for stress-induced de-repression in human cells (Bhattacharyya et al., 2006; Gebert and MacRae, 2019). Recently, Mergner et al. (2020) conducted multi-omics analysis composed of the transcriptome, proteome, and phosphoproteome, to each organ of the whole plant in *Arabidopsis*, and the pollen omics data suggested a low protein-to-mRNA ratio compared to other adult plant tissues. Although it is not yet clear, this low ratio might reflect the hypothesis that the accumulated mRNAs are under translational repression. In rice, a proteome analysis of sperm cells identified approximately 2,000 expressed proteins (Abiko et al., 2013), and a recent rice sperm cell transcriptomic study verified that more than 18,000 genes are transcribed (Rahman et al., 2019). In the maize egg cell, genes encoding proteins for translation are up-regulated (Chen et al., 2017), possibly supporting the active translation of paternally carried-over transcripts in the zygote right after fertilization. Nevertheless, asymmetric division and cell elongation are generated by parent-of-origin effects responsible for the YDA-MAPK cascade and SSP is paternally contributing to it. Future verification will be necessary to reveal whether transcripts other than *SSP* are under translational repression in sperm cells and to elucidate the significance and mechanism of the paternal translational repression in sexual reproduction and the transport of transcripts via plasmogamy.

## Conclusion

The sperm cytoplasm, although significantly reduced, plays a major role in the post-plasmogamic events in the flowering plant zygote. Further investigations of the molecular relationship between Ca^2+^ and the zygotic events, such as cell wall formation and karyogamy (Kranz et al., 1995; Ohnishi et al., 2019), will reveal what exactly is initiated and/or facilitated by the fluctuation in ion amount caused by sperm entry. On the other hand, the observed acceleration of inward movement of the F-actin meshwork after sperm entry in rice early zygotes appears to be independent from the cytosolic Ca^2+^ level (Ohnishi et al., 2019). This implies the presence of factors being carried in other than those regulating Ca^2+^. The YDA-MAP signaling cascade in the *Arabidopsis* zygote is initially activated by paternal SSP, and the *SSP* mRNAs are translationally repressed in the sperm cell and delivered into the egg cell (Bayer et al., 2009). Transcripts from the paternal genome are detected in the *Arabidopsis* zygote (Zhao et al., 2019), and it is not yet clear how many paternally derived transcripts, like *SSP* mRNA, are brought to the zygote and regulate zygote dynamics and subsequent development. Very recently, *Arabidopsis* sperm RNA-seq data has been published (Borg et al., 2020). Together with these omics data and fine-scale imaging, *in vitro* and semi *in vivo* fertilization systems could reveal plasmogamic paternal contributions to the progress of early zygotic development in flowering plants.

## Author Contributions

YO and TK summarized the current investigation and its recent developments in the studies of plant zygotic development, especially plasmogamic paternal contributions via an entry of sperm contents. YO prepared the draft and figures of the mini review. TK summarized and conducted the manuscript preparation and proof trading.

## Conflict of Interest

The authors declare that the research was conducted in the absence of any commercial or financial relationships that could be construed as a potential conflict of interest.
